# Supplementary material for: Family Involvement Training for Staff and Family Caregivers: Case Report on Program Design and Mixed Methods Evaluation
Source: Healthcare (Basel). 2024 Feb 22;12(5):523. doi: 10.3390/healthcare12050523 (PMC10930910; doi:10.3390/healthcare12050523)
Supplement: Supplementary file 1 [file healthcare-12-00523-s001.zip › healthcare-2844769-supplementary.pdf]

Supplementary Files

Supplementary File S1: Survey questions for healthcare providers

1. What is your position in your current employment?
2. How did you hear about the Family Involvement Program?
3. Did you receive education and training about the Family Involvement Program?
4. How satisfied are you with the Family Involvement Education?
5. What feedback can you provide for improving the family involvement program?
6. How satisfied are you with your working relationship with families?
7. How important do you think the Family Involvement Program is in improving resident's quality of life?
8. Why or why do you not think the Family Involvement Program is important in improving resident's quality of life?
9. How important to you think the Family Involvement Program improves resident's care?
10. Why or why do you not think the Family Involvement Program is important in improving resident's care?
11. Do you feel that the Family Involvement Program has strengthened the relationships between families and staff? How?
12. What changes would you recommend to improve the Family Involvement Program?
13. Is there anything else that you would like to add or share about the Family Involvement education or involving family caregivers in resident's care?

Supplementary File S2: Survey questions for family caregivers

1. Relationship to resident
2. Are you aware of the Family Involvement Program at Youville?
3. How important do you think the family involvement program is in improving residents' quality of life
4. How important do you think the family involvement program is in improving residents' care?
5. Do you think involving yourself in care opportunities is important?
6. Do you participate in care opportunities with your loved one?
7. Do you feel supported by staff to carry out these care opportunities?
8. How satisfied are you with your level of involvement in your family member's care?
9. Do you feel the Family Involvement Program improves family and staff relationships?
10. Do your feel there are care opportunities that you would like to know more about?
11. Have you been given an opportunity for more training?
12. Are you interested in education regarding care opportunities?
13. Anything else about your experience with family involvement?

Supplementary File S3: Interview questions for family caregivers

Qualitative Semi-Structured Interviews with Family Caregivers who have taken the Family Involvement Education

Note to research ethics. Methodology: We are using Sally Thorne's Interpretive Description Methodology[1, 2] to guide interviews and interview analysis. We will review the interview transcript

and adjust the interview guide as we learn how research participants make sense of their experiences and we make analytic sense of their meanings and actions.

Introduction:

Thank you for taking part in this interview.

My name is [Name] [and something about yourself]. The study team is led by Dr. Jasneet Parmar from the University of Alberta. Dr. Parmar is a family and care of the elderly physician, so she understands healthcare and works with family caregivers. However, there is little research on how to support family involvement and the impacts of family involvement on family caregivers.

If doing the interview on ZOOM or Phone. We are seeking your verbal consent. Have you received and read the information letter? [If yes proceed, if not read the information letter]

I just want to confirm that you understand:

- That you have read the above information and have had anything that you do not understand explained to you to your satisfaction.
- That you will be taking part in a research study.
- That you may freely leave the research study at any time.
- That you do not waive your legal rights by being in the study
- That the legal and professional obligations of the investigators and involved institutions are not changed by your taking part in this study.

Do I have your verbal consent to proceed with the interview?

Overview:

Alberta's family caregivers provide about 15-30% of the care to residents in supportive living and long term care. However, we know very little about the impacts of educating family caregivers about family involvement in long-term care. We realize you are busy, and we appreciate your time. We expect the interview will take about 45 to 60 minutes.

Anonymity:

If there are any questions or discussions that you do not wish to answer or participate in, you do not have to do so.

I would like to record the interview. The recordings will be kept in an encrypted folder on a password-protected University of Alberta computer safely in a locked facility until they are transcribed word for word. Any information that would allow you or your work setting to be identified will be removed from the transcriptions.

Despite being recorded, I would like to assure you in any reports or academic articles, you will not be identified. May I tape the discussion to facilitate its recollection? (If yes, switch on the recorder).

Guiding questions for participant:

1. **Tell me a bit about your experience with family involvement education?**
   Probes:

   - Why is family involvement in Long-term care important to you? To your family member?
2. **Did the family involvement education make a difference to you? How? Change anything for you?**
   - Confidence? Comfort?
   - Relationship with your family member
   - Your Quality of life
   - Relationship with staff
3. **How did the family involvement education make a difference to your family member?**
   - Your family members' quality of life
     - Can you give me some examples Or Can you tell me a story of how you noticed a difference in your family member's quality of life?
   - Your family members' quality of care
     - Can you tell me a story of how you noticed a difference in your family member's quality of care?

4. **How did the Family Involvement affect your relationships with staff?**
   Probes:

   - Can you give me an example or particular instance of how your relationship with staff changed? (encourage stories of involvement and relationships with staff)
   - What was it about the education that changed relationships with staff?

5. **Did you experience any barriers to your participation in the family involvement project?**
6. **How might we improve the family involvement education/ project?**
7. **I have asked you lots of questions, is there anything that you would like to ask me or tell us?**

Thank you very much. This has been incredibly helpful. Would you like a copy of the report when we are done?
